# Non‐conventional method of pontic site preservation with laser‐assisted bone regeneration

**DOI:** 10.1002/ccr3.4483

**Published:** 2021-07-16

**Authors:** Soher Nagi Jayash, Ghassan Habash, Mudar Kamal

**Affiliations:** ^1^ School of Dentistry University of Birmingham Birmingham UK; ^2^ Deanship of Scientific Research Al‐Quds University Al‐Quds Palestine; ^3^ University of Health Sciences Lahore Lahore Pakistan; ^4^ Prosthodontic Department Faculty of Dentistry Al‐Quds University Al‐Quds Palestine

**Keywords:** implant, laser treatment, pontic site preservation

## Abstract

This report presents an alternative method to the removal of failing implant and using a bone graft to preserve ridge which needs several months to heal and it is a costly technique. The pontic site was preserved by covering the failing implant with connective tissue and laser‐assisted peri‐implant defect regeneration.

## Graphical abstract

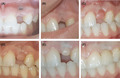

## 1 INTRODUCTION

Many techniques aim to augment ridge after extraction and to treat failing implants. The implant may need to remove and use bone graft for ridge preservation. This case report describes a non‐conventional technique to preserve the pontic site by covering an implant with connective tissue grafts and laser‐assisted treatment.

Resorption of the alveolar ridge begins immediately post‐extraction or removal of the implant, and this is more pronounced on the buccal aspect and may lead to loss of 56% of the residual ridge.^1^ Positioning a pontic restoration at a missing tooth site needs to make aesthetic harmony between the restoration and the alveolar ridge. Most instances require management of these extraction sites either by preventing tissue loss by ridge preservation techniques or by augmenting the already collapsed tissues by bone augmentation, soft tissue augmentation, or a combination.^2^, ^3^

Spontaneous exposure of the implant cover screw is a frequent complication. It could be due to extremely thin tissues surrounding the implant, trauma causing atrophy or necrosis of the mucosa, or to loosening of the cover screw resulting displacement of the overlying soft tissues. Perforations of the mucoperiosteum may lead to inflammation, damage to the peri‐implant mucosa, and bone loss by bacterial plaque formation. This may be treated by elevating a flap to cover the mucoperiosteal perforation with or without grafts or membranes.^4^

In the case of dental implant failure, the implant may need to be removed and may require a bone graft which takes several months to heal before placing a new implant. The use of laser helps to eliminate microbes as well as the faster and better healing of soft and hard tissues. Moreover, low‐level laser therapy (LLLT) reinforces the revitalization process, enhances the healing of injured tissues, and has shown a promising therapeutic effect in the treatment of peri‐implantitis.^5^, ^6^ This case report describes a case with an ill‐fitting crown on the failing implant and mucoperiosteal perforation being treated by covering the implant with a connective tissue graft and laser treatment and finally restored by an all‐ceramic minimally prepared resin‐bonded bridge. The pontic of the bridge was used to shape the augmented area resulting in better tissue architecture.

## 2 CASE REPORT

### 2.1 Methods

**FIGURE 1:**
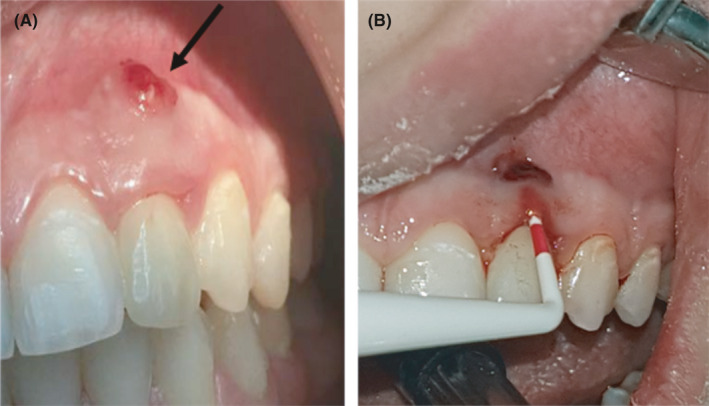
(A) Buccal fistula after implant placement. (B) Buccal pocket depth around the implant

**FIGURE 2:**
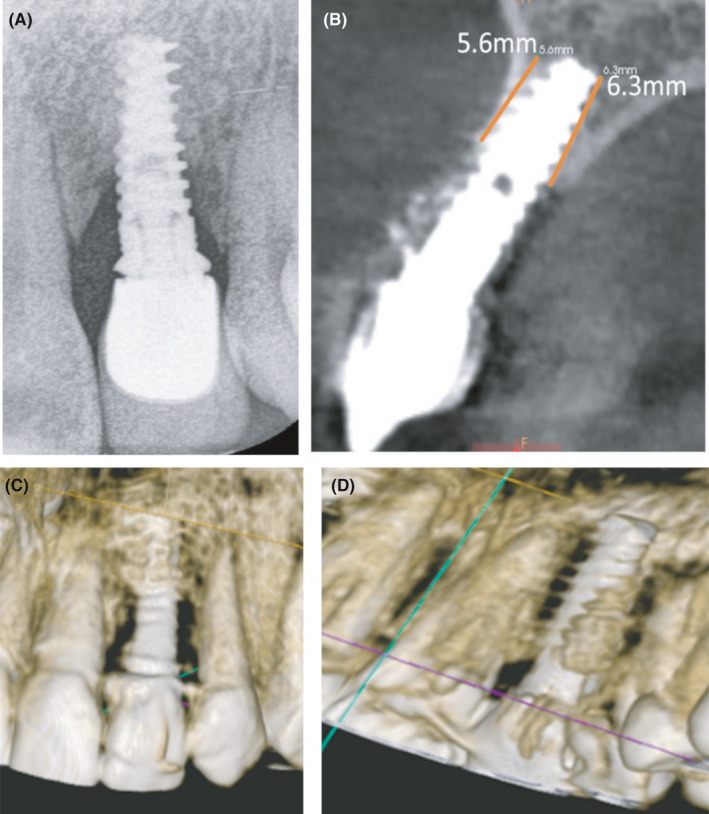
(A) Intraoral periapical radiograph of the implant site. (B) The CBCT imaging of implant site showing bone level in sagittal view. 3D composition of the implant site in buccal view (C) and palatal view (D)

A 20‑year‑old female patient with good general health referred to the dental clinic with a chief complaint of a poorly fitted crown associated with swelling in buccal mucosa that developed after implant placement in early 2017 in upper left congenital missing lateral and referred to us 9 months after fistula creation. Clinical examination revealed the buccal fistula, pus discharge around the implant, and ill‐fitting crown with excess cement (Figure 1A). Buccal pocket depth was 6 mm and extending from the sulcus to the fistula which meant there is no attachment at the buccal site with the implant (Figure 1B). Periapical radiograph and cone beam computed tomography (CBCT) scan revealed bone resorption in the mesial and distal surfaces and absence of the buccal plate and the lingual plate up to two‐third of implant, making the prognosis for retreatment poor. The alveolar bone‐height measurements from CBCT images were 5.6 and 6.3 mm in buccal and lingual sides of the implant, respectively (Figure 2A–D). There were two treatment options: First option was to remove the implant followed with guided bone regeneration and then followed by implant placement. However, there was not enough soft tissue to cover the bone graft and the vertical bone augmentation has a less predictable prognosis. The second option was adopting a non‐conventional method of pontic site preservation by covering the implant with connective tissue graft and LLLT and the space restored by a minimally prepared resin‐bonded bridge. The second option was planned following patient consent for the treatment, and ethical approval was obtained from Al‐Quds University.

Non‐surgical debridement was done after achieving adequate local anesthesia by using manual titanium, plastic curettes, and prophy.

**FIGURE 3:**
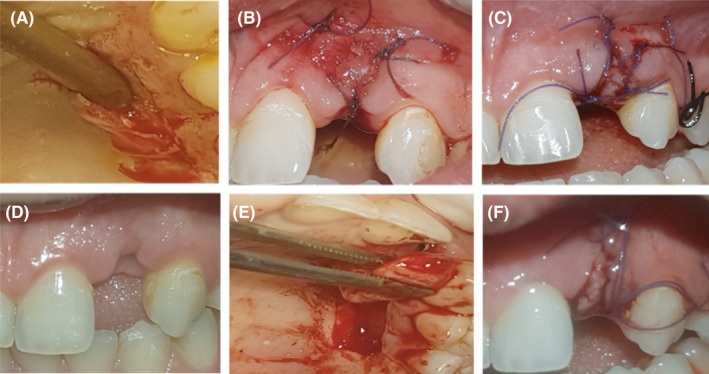
(A) Harvested connective tissue graft by trap‐door technique. (B) Connective tissue graft sutured in buccal surface of the implant. (C) Connective tissue graft sutured in occlusal surface of the implant. (D) Implant site at 2‐month follow‐up after connective tissue graft sutured in the occlusal surface. (E and F) Full‐thickness rotational pedicel flap with suture

After 2‐month follow‐up, laser cold tip 1 W pulsating mode was used to decontaminate the implant surface with application of titanium brush and minocycline. The patient was prescribed antibiotic therapy that is Augmantine 500 and 250 mg Flagyl for 1 week and 0.12 chlorhexidine mouthwash for 2 weeks. The crown was removed, connective tissue graft was performed for increasing the bucco‑palatal dimensions, and LLLT was applied immediately after surgery and 1, 2, and 4 weeks after surgery. At 3‐month follow‐up, connective tissue graft was performed to cover the implant and increase vertical dimension (Figure 3).

### 2.2 Connective tissue graft surgery harvested through trap‐door technique

The connective tissue graft was harvested from the palate using the trap‐door technique^7^ using a horizontal incision 3–4 mm away from the gingival margin with two vertical incisions on either end of the first incision creating a door. The door is then undermined and opened using a sharp dissection, the underlying connective tissue is then harvested using a periosteal elevator, and the door was then sutured and the connective tissue was transferred to the buccal surface of the implant and sutured (Figure 3A–C).

At 2‐month follow‐up, unfortunately, there was no improvement in the vertical dimension because the harvested connect tissue was thin and not enough to increase the vertical site (Figure 3D). Thus, a full‐thickness rotational pedicel flap was performed (Figure 3E,F). Then, pontic site development was planned after complete healing of the occlusal tissue.

### 2.3 Full‐thickness rotational pedicel flap

The full‐thickness pedicle flap was raised 3 mm beyond the mucogingival junction with two horizontal incisions and one vertical incision. The flap was placed in a position to cover the occlusal surface of the implant and sutured.

The patient was recalled at 1, 2, and 4 months. Healing was uneventful with minimal postoperative discomfort in relation to the treated site. After 4 months, all‐ceramic resin‐bonded bridge (lithium disilicated ceramic, IPS e‐max Press^®^, Ivoclar Vivadent) was retained on teeth number 2.1 and 2.3. The pontic design was a modified ridge lap, and the light cure resin cement (Choice 2^®^, Bisco) was used for cementation. The patient was reviewed after 1 year.

### 2.4 Laser therapy

Laser therapy was applied using a cold tip (1 W) with pulsating mode to keep moving up and down for 30 s on the surgical area. The surgical area was irradiated using 810 nm low‐level laser therapy (Diode laser, QuickLase), in non‐contact pulsating wave mode (0.1 W) in an apicocoronal, back and forth directions for 45 s.

## 3 RESULTS

**FIGURE 4:**
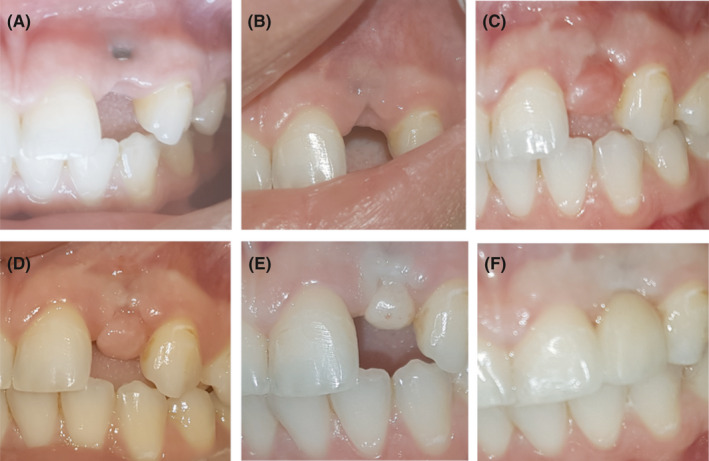
(A) Clinical photograph of implant site 2 months after non‐surgical debridement. (B) Clinical photograph of the buccal site of the implant after connective tissue graft at 3‐month follow‐up. Clinical photograph of implant site after full‐thickness rotational pedicel flap after 1 month (C), 2 months (D), and 4 months (E). (F) Clinical photograph after placement of the final restoration

At 2‐month follow‐up after non‐surgical debridement, there was no inflammation in the lesion area and part of the implant screw exposed (Figure 4A). This was followed by a connective tissue graft to cover the implant and increase the bucco‐palatal width. At 3‐month follow‐up, examination revealed an increase in the bucco‐palatal width of the ridge and screw of the implant is completely covered with buccal tissue. Also, a depression in the occlusal area of the ridge was observed (Figure 4B). To cover this depression and increase the vertical dimension, a full‐thickness rotational pedicel flap was done. At 1‐, 2‐, and 4‐month follow‐up, healing in the pontic site was successfully sculpted to accommodate the final restoration. An eventual positive outcome and ridge high was maintained (Figure 4C–F).

**FIGURE 5:**
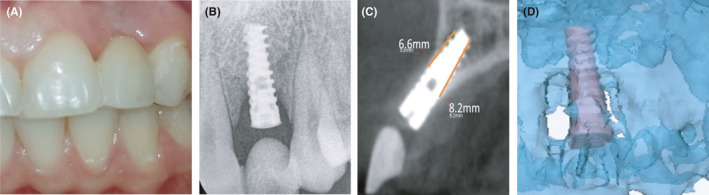
(A) Clinical photograph of implant site with a resin‐bonded bridge at 1‐year follow‐up. (B) Intraoral periapical radiograph of implant site at 1‐year follow‐up. (C) The CBCT imaging of implant site showing bone level in sagittal view. (D) 3D volume matching of implant site before treatment and 1 year after treatment shows new bone formation in mesial and distal sites of implant

One year follow‐up revealed a pleasing aesthetic treatment outcome. The pontic sites' tissues were clinically healthy and the radiological examination showed bone regenerated around the implant (Figure 5A,B). The alveolar bone‐height measurements from CBCT images were 6.6 and 8.2 mm in buccal and lingual of implant, respectively (Figure 5C). 3D volume matching of bone in implant site before treatment and after treatment showed new bone formation in mesial and distal sites of the implant (Figure 5D).

## 4 DISCUSSION

Preserving soft tissues and prevention of bone collapse following tooth extraction or removal of the failed implant are challenges. The bone preservation procedure is employed at the time of the failed implant in aesthetically demanding areas, and soft tissue augmentation procedures may be needed after the healing of the implant site to optimize the aesthetics. Various soft tissue procedures for improving ridge deformities are described including subepithelial connective tissue graft that is used to preserve tissue color and the texture of the underlying mucosa and provided greater soft tissue volume, resulting in better aesthetics.^7^ The use of LLLT in periodontics has allowed the periodontists to achieve better clinical results.^8^, ^9^ LLLT produces sterilization of tissue surface leading to decreased bacteremia, edema, swelling, and scarring.^10^ Additionally, LLLT accelerates wound healing by stimulating epithelization and regeneration of human and animal tissue.^11^ Subsequently, LLLT may show significant improvement in the predictability and stability of clinical outcomes for tissue defects. Loss of buccal soft tissue in dentistry is one of the major aesthetic concerns that can be treated using soft tissue graft techniques, but the predictability and stability of the outcomes are debatable. LLLT adjunct to connective tissue graft depicted a significant improvement in the predictability and stability of tissue healing outcomes compared with connective tissue graft alone.^12^ Thus, in this case report, LLLT adjunct to connective tissue grafts was applied to increase sufficient labiolingual width and apicocoronally thickness that were required for housing the pontic.

Following removal of the failed implant, the bone resorbs and may lead to the collapse of residual bucco‐palatal tissues. Treatment of ridge defects following removal of the implant may require extensive surgical intervention before definitive restorative treatment. This may involve guided bone regeneration techniques using bone and/or bone substitutes that may lead to increase hard tissue, but these techniques have limitations including increased morbidity, technique sensitivity, increased costs, the difficulty of access to materials, and scarring from the ridge augmentation procedure.^2^ Many clinical studies submerge root to preserve the ridge^3^, ^13^, ^14^; however, this is the first clinical study used to cover the failing implant with the connective tissue graft.

Thus, implant submergence is introduced as a technique to preserve alveolar ridge volume and reported successful outcomes in the development of the ridge to accommodate a pontic restoration. Moreover, bone regeneration around the implant is observed by an increase of 2–3 mm bone height after 1 year of treatment. This is proving the effectiveness of LLLT in bone healing around the implant without using a bone graft. This technique involves the covering of the failing implant with connective tissue grafts with laser assessed treatment, and the follow‐up observation showed no complication after treatment and excellent aesthetic result. It is advised that future clinical studies be carried out that include comparing the preservation of tissues using this technique to the control that removes the implant and uses bone graft.

## 5 CONCLUSION

The clinical case report demonstrated covering an implant with laser treatment as a therapy for the development of pontic sites with excellent soft tissue support at 1‐year follow‐up. Thus, covering failing implant with connective tissue graft used in conjunction with low‐level laser therapy is recommended to enhance tissue healing and increase bucco‐palatal width and height.

## CONFLICT OF INTEREST

None declared.

## AUTHOR CONTRIBUTION

GH: performed surgical, laser treatments and collected the data. MK: performed the restorative treatment. SNJ: wrote the manuscript and analyzed the data.

## ETHICAL APPROVAL AND CONSENT TO PARTICIPATE

Patient consent to participate is signed before starting the treatment, and ethical approval is submitted as supplementary 1.

## Data Availability

All supporting data are available.
